# Associations Between Adolescents’ Social Re-orientation Toward Peers Over Caregivers and Neural Response to Teenage Faces

**DOI:** 10.3389/fnbeh.2019.00108

**Published:** 2019-05-24

**Authors:** Michele Morningstar, Connor Grannis, Whitney I. Mattson, Eric E. Nelson

**Affiliations:** ^1^Center for Biobehavioral Health, Nationwide Children’s Hospital, Columbus, OH, United States; ^2^Department of Pediatrics, The Ohio State University, Columbus, OH, United States

**Keywords:** adolescence, social development, peers, faces, social brain, relationships

## Abstract

Adolescence is a period of intensive development in body, brain, and behavior. Potentiated by changes in hormones and neural response to social stimuli, teenagers undergo a process of social re-orientation away from their caregivers and toward expanding peer networks. The current study examines how relative relational closeness to peers (compared to parents) during adolescence is linked to neural response to the facial emotional expressions of other teenagers. Self-reported closeness with friends (same- and opposite-sex) and parents (mother and father), and neural response to facial stimuli during fMRI, were assessed in 8- to 19-year-old typically developing youth (*n* = 40, *mean* age = 13.90 years old, *SD* = 3.36; 25 female). Youth who reported greater relative closeness with peers than with parents showed decreased activation in the dorsolateral prefrontal cortex (dlPFC) during stimulus presentation, which may reflect lessened inhibitory control or regulatory response to peer-aged faces. Functional connectivity between the dlPFC and dorsal striatum was greatest in older youth who were closer to peers; in contrast, negative coupling between these regions was noted for both younger participants who were closer to peers and older participants who were closer to their parents. In addition, the association between relative closeness to peers and neural activation in regions of the social brain varied by emotion type and age. Results suggest that the re-orientation toward peers that occurs during adolescence is accompanied by changes in neural response to peer-aged social signals in social cognitive, prefrontal, and subcortical networks.

## Introduction

Adolescence is often considered a second sensitive period of development, because it is a time when dramatic changes in emotion, cognition, and behavior take place (Crone and Dahl, 2012). Due in part to fluctuations in adrenarcheal and gonadal hormones during the teenage years (Forbes and Dahl, 2010; Byrne et al., 2017), marked structural and functional development occurs in numerous brain networks related to motivation (Forbes and Dahl, 2010; Goddings et al., 2012; Scherf et al., 2013), executive function (Crone, 2009; Ordaz et al., 2013; Satterthwaite et al., 2013), and social cognition (Blakemore, 2008). The neural maturation of detection, affective, and cognitive regulation systems in the brain are thought to help guide the processing of increasingly complex socio-emotional stimuli during a period where teenagers begin to engage with broader social networks outside of their family environment [see social information processing network (SIPN) model; Nelson et al., 2005; Nelson et al., 2016].

In many contexts, adolescence is the apex of an inverted U-shaped maturational curve for affective or motivational responses, but represents only an intermediary point in the linear trajectory of higher cognitive functions (Casey et al., 2010a; Smith et al., 2014). For example, compared to children or adults, adolescents show heightened response to both threatening and rewarding stimuli in areas associated with motivational aspects of affective experience, such as the amygdala, striatum, anterior insula, and anterior cingulate cortex (Casey and Jones, 2010; Moore et al., 2012; van Duijvenvoorde et al., 2014; Smith et al., 2015, 2018; Braams et al., 2016; Guyer et al., 2016). However, prefrontal regions associated with cognitive regulation and the canalization of motivational responses continue to develop into adulthood, as does their neuromodulatory influence on subcortical affective systems (Steinberg, 2005; Crone, 2009; Casey et al., 2010b; Nelson and Guyer, 2011). In adolescence, immature prefrontal regulation of reward- and affect-related responses may be contributing to the heightened salience attributed to peers and other emotional stimuli (Guyer et al., 2009; Nelson and Guyer, 2011; Schriber and Guyer, 2016). Potentiated motivational responses to developmentally relevant stimuli, such as social cues from other youth, may be in fact an important mechanism that guides increases in engagement with peers (Larson and Richards, 1991; Nelson et al., 2016) during the teenage years.

Achieving independence from caregivers and integrating with peer networks is one of the more dramatic transitions that occurs during adolescence. Indeed, across both cultures and species, puberty is accompanied by a marked shift in social landscape, whereby individuals spend greater amounts of time with peers and less time in proximity to primary caretakers (Nelson et al., 2005; Forbes and Dahl, 2010; Crone and Dahl, 2012). This social re-orientation is likely encouraged by changes in emotional responses elicited by salient social cues, which promote adolescents’ behavioral shift toward peers. For instance, though parents are the primary source of emotional support for 9- to 10-year-olds, youth’s dependency on parents declines from early to mid-adolescence – with same-sex friends becoming the main source of support and intimacy for 15- to 16-year-olds (Hunter and Youniss, 1982; Furman and Buhrmester, 1992; Rice and Mulkeen, 1995; Lieberman et al., 1999; De Goede et al., 2009). At a physiological level, the presence of mothers has been found to buffer the cortisol stress response and modulate amygdala reactivity in children, but not in adolescents (Gee et al., 2014; Hostinar et al., 2015). Thus, the social re-orientation of adolescence is accompanied by a reconfiguration of the salience of social cues (Spear, 2000; Ladouceur, 2012), with peer-aged social signals becoming increasingly important relative to those of parents. This in turn promotes behavioral engagement with peers and associated social learning (Nelson et al., 2016), whereby teenagers adapt to the specific behavioral norms of new peer groups outside of the family environment to gain social acceptance (O’Brien and Bierman, 1988; Lamblin et al., 2017).

Changes in the valuation and salience of peers during adolescence may also guide the development of increasingly specialized neural networks for the processing of social information (Spear, 2000; Casey et al., 2010a; Nelson et al., 2016). Indeed, the maturation of perceptual and socio-emotional networks is thought to be guided by experience (Johnson et al., 2009; Leppanen and Nelson, 2009; Crone and Dahl, 2012; Pfeifer and Blakemore, 2012; Scherf and Scott, 2012; Dahl et al., 2018). In infancy, emotion and attention networks are attuned to salient social stimuli (Carver et al., 2003; Leppanen and Nelson, 2009) – such as caregiver faces and voices (Querleu et al., 1984; Bushneil et al., 1989; Carver et al., 2003; Tottenham et al., 2012) – when critical maturational changes are taking place within perceptual networks. Emotion-guided attention to caregivers is thought to play an important role in shaping the neuronal responses to these stimuli, which persist throughout subsequent developmental stages (Sugita, 2008; Leppanen and Nelson, 2009; Beauchemin et al., 2010; Nakato et al., 2011; Werker and Hensch, 2015). Similarly, when peers are gaining in emotional importance during adolescence, functional maturation is taking place in many brain areas involved in social cognition processes, such as the orbitofrontal and ventral lateral prefrontal cortex, amygdala, and posterior superior temporal sulcus (for reviews, see Paus, 2005; Blakemore, 2008; Burnett et al., 2011). These developmental changes coincide with increases in social cognition abilities, including mentalizing and the recognition of facial emotional expressions (Steinberg, 2005; Blakemore and Mills, 2014; Kilford et al., 2016; Foulkes and Blakemore, 2018). Therefore, the adolescent transition toward peers is likely to be mediated by relative shifts in emotion and motivation, and may promote social learning by guiding functional maturation of emerging social cognitive networks in the brain. However, though extensive work has examined both teenagers’ changing relationships with peers and parents (e.g., Furman and Buhrmester, 1992; Steinberg and Morris, 2001) and their neural responses to socio-emotional stimuli (e.g., Burnett et al., 2009, 2011), the association between adolescents’ social orientation toward peers versus parents and concomitant brain activation in response to peer-aged social stimuli has not been investigated.

The current study examines how age-related changes in self-reported emotional closeness to peers vs. parents in 40 typically developing adolescents (aged 8 to 19 years old) are associated with differential neural activation to peer-aged facial expressions of emotion. The SIPN model suggests that salience-related response in limbic structures may guide approach or avoidance behaviors toward developmentally relevant stimuli, such as socio-emotional cues from other teenagers (Nelson et al., 2005, 2016; Leppanen and Nelson, 2009). Thus, as youth re-orient toward their peers during adolescence, the facial non-verbal expressions of other teenagers are likely to be more salient and rewarding (e.g., Wright and Stroud, 2002; Chein et al., 2011; Picci and Scherf, 2016). For example, choosing to approach pictures of friends (over those of familiar peers or celebrities, using a joystick) has been associated with greater amygdala, hippocampus, nucleus accumbens, and ventral medial prefrontal cortex activation in adolescents (Güroğlu et al., 2008), suggesting a valuation response to peers at this age. Similarly, adolescents showed more activation to videos of unfamiliar teenagers’ emotions than of their parents’ in mentalizing (temporal-parietal junction, posterior superior temporal sulcus) and subcortical emotion processing regions (ventral striatum, amygdala, and hippocampus; Saxbe et al., 2015). As such, we hypothesized that youth who reported greater closeness with their peers (compared to their parents) would also show increased neural response to peer-aged faces in reward- or affect-related regions (e.g., amygdala, ventral striatum, hippocampus) and social processing areas (e.g., temporal-parietal junction) of the brain.

In conjunction, response within cognitive-regulatory regions of the brain may be lower. Theories of adolescent neurodevelopment (including the SIPN and the dual-systems model; Nelson et al., 2016; Shulman et al., 2016) suggest that the motivational influence of peers on behavior during the teenage years may be due in part to insufficient prefrontal regulation of subcortical responses. As such, frontal cortical regions associated with cognitive regulation may not be as highly engaged in youth who show evidence of social re-orientation toward peers. However, there is likely to be change in the relative engagement of both the affective and cognitive-regulation node with peer-aged stimuli across development (Nelson et al., 2005). Further, given evidence of age-related changes in both peer relationships (Larson and Richards, 1991; Steinberg and Morris, 2001; Foulkes and Blakemore, 2018) and face processing (Cohen Kadosh and Johnson, 2007; Cohen Kadosh et al., 2011, 2013; Moore et al., 2012; Pfeifer and Blakemore, 2012), we expected that the association between peer experiences and neural response to teenagers’ facial expressions of emotion would vary as a function of age from late childhood to late adolescence.

## Materials and Methods

### Participants

The study sample included 40 typically developing youth (25 female) between the ages of 8 and 19 years old (*M* = 13.90, *SD* = 3.36). Because the timing of social re-orientation differs between individuals, we included participants within a broad age range to capture variation in social engagement across childhood and adolescence. Participants were recruited through a digital flyer distributed via email to employees of a large Midwestern children’s hospital. Exclusion criteria included severe cognitive impairment and the presence of conditions or devices contraindicated for magnetic resonance imaging (MRI; e.g., braces, retainer, pacemaker), assessed via a metal screening form. Self-report of race indicated that 67.5% of the sample was Caucasian, 17.5% was Black or African American, and 15% was multiracial or of other ethnicities. Participants provided written assent or consent. Parents of participants younger than 18 provided written parental consent for their child’s participation. All procedures were approved by the hospital Institutional Review Board.

### Measures

#### Closeness to Peers and Parents

Closeness to peers and parents was assessed using the Network of Relationships Inventory – Relationship Qualities version (NRI) questionnaire (Furman and Buhrmester, 2009). Participants answered 30 questions about different aspects of their relationships with 6 people in their lives: best same-sex friend, best opposite-sex friend, boy/girlfriend, sibling, mother, and father (Since not all participants in our sample had a boy/girlfriend or a sibling, these relationships were excluded from further analyses). Relationship features are rated on a 5-point scale, from 1 = “never or hardly at all” to 5 = “always or extremely.” The subscales for companionship, intimate disclosure, satisfaction, emotional support, and approval were averaged to create a “closeness score” for each relationship (Furman and Buhrmester, 2009). To assess relative closeness in different relationship types (e.g., peers compared to parents), we generated a closeness score for peers (average closeness with best same-sex and best opposite-sex friend; α = 0.93) and a closeness score for parents (average closeness with mother and father; α = 0.94) for each participant. Same- and opposite-sex friends were merged to obtain the peer closeness score, since the majority of youth report having meaningful opposite-sex friendships at this age (Kuttler et al., 1999). A Relative Closeness score was then obtained by subtracting closeness with parents from closeness with peers: positive values of Relative Closeness indicate greater closeness with peers than with parents, and negative values indicate greater closeness with parents than with peers.

#### Neural Response to Facial Expressions

Participants’ neural response to facial stimuli was assessed in the context of a facial emotion recognition (ER) task. As part of a larger study, youth were presented with pictures of adolescents’ facial expressions (conveying anger, fear, happiness, sadness, or neutral) and asked to identify the intended emotion from the above five labels while undergoing functional magnetic resonance imaging (fMRI). Facial stimuli were selected from the National Institute of Mental Health’s Child Emotional Faces Picture Set (NIMH-ChEFS; Egger et al., 2011). Forty-five faces were produced by female adolescents (nine actors) and 45 by male adolescents (six actors), for a total of 90 facial expressions. Six faces were selected for each of the five emotional expressions. Within these six faces, three faces had their eyes averted away from the participant, and 3 faces had a straight eye gaze. The same child provided both the straight- and averted-gaze version of a stimulus. The stimuli were selected from the full dataset based on expression quality, judged by two research assistants (see Supplementary Table 2 in Supplementary Materials).

Following training in a mock scanner, participants completed the ER task in the MRI scanner. Each trial was comprised of stimulus presentation (1 s in duration) followed by a 5-s response period. Participants viewed a computer monitor at the head of the magnet bore via a mirror attached to the head coil and responses were recorded using a Lumina handheld response device inside the scanner. Stimuli were presented in an event-related design with a jittered inter-trial interval between 1 and 8 s (mean 4.5 s). A fixation cross was visible during the inter-trial interval and a pictogram of response labels was shown during the response period. The task was split into three runs of 30 faces, each lasting approximately 6 min. Each run contained a pseudorandomized order of faces that included a balanced number of stimuli per emotion type. Runs were presented in random order.

### Image Acquisition and Processing

Magnetic resonance imaging data were collected on two Siemens 3 Tesla scanners running identical software, using standard 32- and 64-channel head coil arrays.^1^ Imaging protocol included three-plane localizer scout images and an isotropic 3D T1-weighted anatomical scan covering the whole brain (MPRAGE). Imaging parameters for the MPRAGE were: 1 mm pixel dimensions, 176 sagittal slices, repetition time (TR) = 2200–2300 ms, echo time (TE) = 2.45–2.98 ms, field of view (FOV) = 248–256 mm. Functional MRI data were acquired with echo planar imaging (EPI) acquisitions, with a voxel size of 2.5 × 2.5 × 3.5–4 mm, and with the phase-encoding axis oriented in the anterior-posterior direction. During fMRI scans, dummy data were collected for 9.2 s while participants watched a blank screen. For fMRI scans, parameters were: TR = 1500 ms, TE = 30–43 ms, FOV = 240 mm.

Echo planar imaging images were preprocessed and analyzed in AFNI, version 18.0.11 (Cox, 1996). Functional images were corrected to the first volume, realigned to the AC/PC line, and coregistered to the T1 anatomical image. The image was subsequently normalized non-linearly to the Talairach template. After normalization, data were spatially smoothed with a Gaussian filter (FWHM, 6 mm kernel). Voxel-wise signal was scaled to a mean value of 100, and signal values above 200 were censored within each functional run. Volumes in which at least 10% of the voxels were considered to be signal outliers or contained movement greater than 1 mm between volumes were censored prior to analysis. Following this procedure, 4.1% of volumes were censored.^2^

### Analysis

#### Relative Closeness With Peers and Parents

A general linear model was computed to examine the association between Relationship Type (within-subjects, two levels: peers vs. parents), Age (between-subjects; continuous, in years), and biological Sex (between-subjects, two levels: male vs. female) on NRI closeness scores. In addition, a regression was performed to examine the association between Age and NRI Relative Closeness scores.

#### Neural Response to Faces

Event-related response amplitudes were first estimated at the subject level. We convolved the hemodynamic response function with a base function that included a combined regressor for the presentation of the facial stimulus (1 s in duration) contrasted to the baseline fixation cross and response period. A regressor for stimulus emotion category (five levels) and nuisance regressors for motion (six affine directions) and scanner drift within the concatenated runs (3rd polynomial) were also included at the subject level. For group-level analyses, the contrast images produced for each participant were fit to a multivariate model (3dMVM in AFNI; Chen et al., 2014) of the effect of Emotion category, mean-centered Relative Closeness, and mean-centered Age on whole-brain activation, with participant Sex as a control variable. Within this model, we computed *F-*statistics for the main effects of Emotion, Age, Relative Closeness, and for the interactions of Relative Closeness × Age and Relative Closeness × Emotion. Cluster-size threshold corrections were estimated with the spatial autocorrelation function of 3dclustsim, based on Montecarlo simulations with study-specific smoothing estimates (Cox et al., 2016), with two-sided thresholding and first-nearest neighbor clustering, at α = 0.05 and *p* < 0.001. The resulting cluster threshold of 27 voxels was applied to the results. Regions were identified at their peak activation point using the Talairach-Tournoux atlas.

## Results

### Relative Closeness With Peers and Parents

**FIGURE 1:**
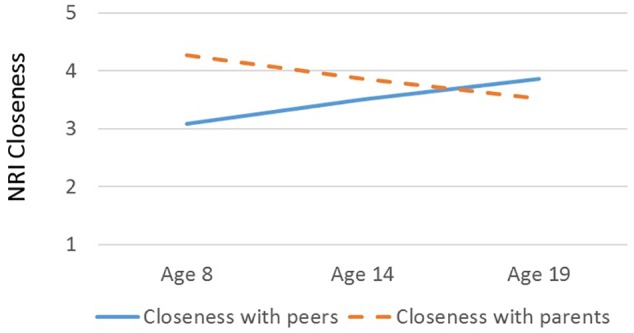
Interaction between age (in years) and closeness scores on the Network of Relationships Inventory. Blue solid line, closeness to peers (same- and opposite-sex friend); orange dashed line, closeness to parents (mother and father). Plotted closeness scores are estimated from marginal means for different relationship types (peers vs. parents) at age 8, age 14, and age 19, with Sex held constant.

**FIGURE 2:**
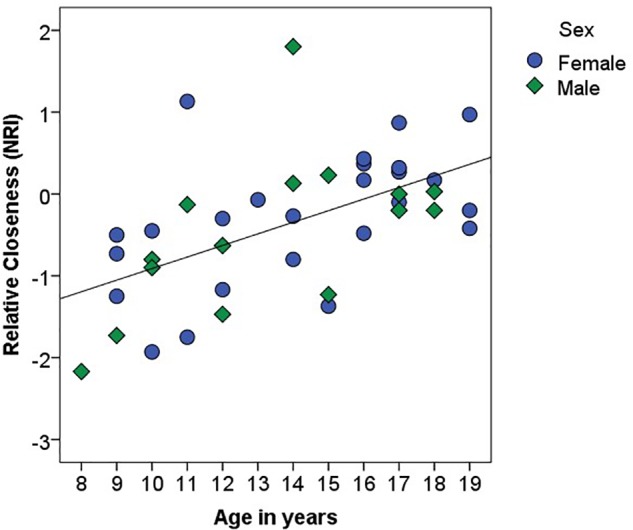
Association between age (in years) and Relative Closeness on the Network of Relationships Inventory (NRI). Relative Closeness = closeness with peers – closeness with parents. Female participants are identified with blue circles; male participants are identified with green diamonds. The black line represents the linear relationship between the two variables (*R*^2^ = 0.31).

There was a main effect of Relationship Type on closeness scores, *F*(1, 37) = 21.21, *p* < 0.001, η^2^ = 0.36, such that participants reported generally greater closeness within their parental than peer relationships (Table 1). However, there was a significant interaction between Relationship Type and Age, *F*(1, 37) = 15.66, *p* < 0.001, η^2^ = 0.30: parameter estimates suggested that closeness with peers increased with age (*B* = 0.07, β = 0.29, *p* = 0.07) and closeness with parents decreased with age (*B* = -0.07, β = -0.47, *p* < 0.01; see Figure 1). There was no main effect of Age (*p* > 0.96), Sex (*p* > 0.60), or interaction between Sex and Relationship Type (*p* > 0.76) on closeness. Further, Age predicted higher Relative Closeness scores, β = 0.55, *t*(38) = 4.09, *p* < 0.001, suggesting that older participants were closer to their peers (over parents) than were younger participants (Figure 2).

**Table 1:** Closeness with peers, closeness with parents, and Relative Closeness scores on the NRI.

		Standard
Relationship type	Mean	deviation	Minimum	Maximum
Closeness to peers	3.52	0.81	1.60	5.00
Closeness to parents	3.87	0.49	2.97	4.67
Relative closeness to peers over	-0.36	0.86	-2.17	1.80
parents (Relative Closeness)


### Neural Response to Faces

**FIGURE 3:**
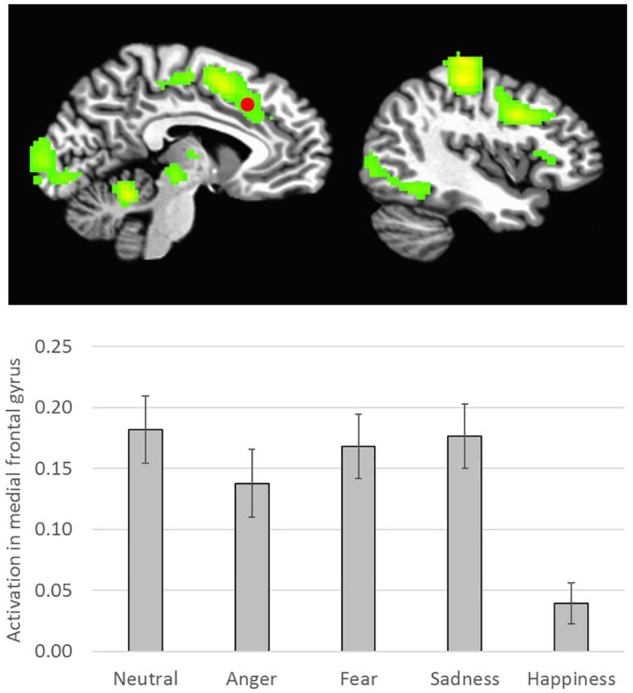
Emotion-specific activation during stimulus presentation (i.e., *F* of Emotion). Clusters were formed using 3dclustsim at *p* < 0.001, with a cluster size threshold of 26 voxels. Brain images are rendered in the Talairach-Tournoux template space. Refer to Table 2 for description of regions of activation. Bar graph represents estimated marginal means for effect of Emotion type on activation in the medial frontal gyrus (marked with a red dot on the brain image), with Sex, Relative Closeness, and Age held constant at the mean. Error bars represent the standard error of the mean.

**FIGURE 4:**
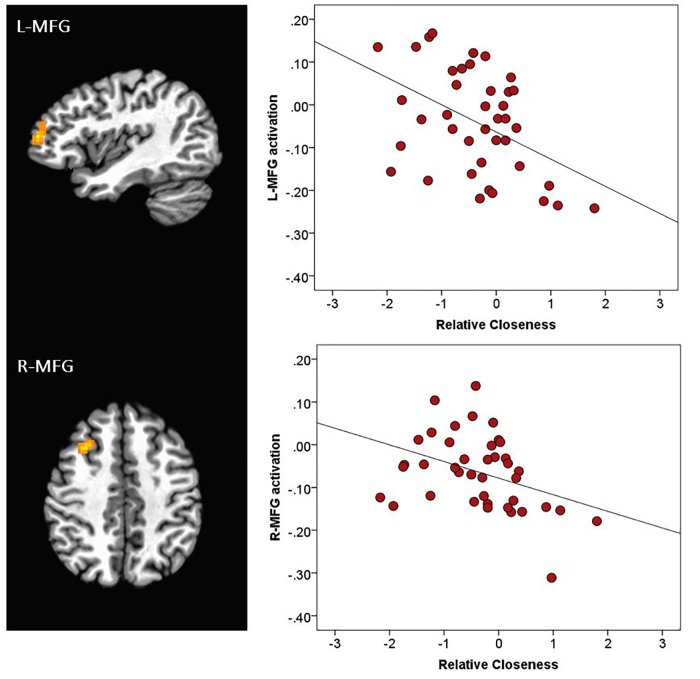
Activation during stimulus presentation associated with participants’ relative closeness with peers over parents. R, right; L, left. MFG, middle frontal gyrus. Clusters were formed using 3dclustsim at *p* < 0.001, with a cluster size threshold of 27 voxels. Brain images are rendered in the Talairach-Tournoux template space. Refer to Table 2 for description of regions of activation. The black line on the scatterplots represents the linear relationship between neural activation and Relative Closeness (L-MFG: *R*^2^ = 0.20; R-MFG: *R*^2^ = 0.15).

There was a main effect of Emotion in several brain areas, including the medial frontal gyrus at midline, right inferior frontal gyrus, right and left insula, right superior temporal gyrus, and right and left temporo-parietal junction (Table 2 and Figure 3). All of these clusters showed a similar emotion-specific pattern, whereby happy faces elicited relatively less activation than the other types of emotional faces (except for a cluster in the right insula, where neutral elicited relatively greater activation than the other emotions). There was no main effect of Age or Sex on brain activation to peer-aged faces. However, there was a main effect of Relative Closeness on activation in the left and right middle frontal gyri (L-MFG; R-MFG), where increased closeness with peers over parents was associated with lessened response to peer-aged faces (Table 2 and Figure 4).

**Table 2:** Effects of Relative Closeness, Age, and Emotion on neural activation to faces.

Effect structure	*F*	*k*	*x*	*y*	*z*	Generalized η^2^	Brodmann area
Relative Closeness							
L middle frontal gyrus (L-MFG)	33.07	46	-39	49	11	0.17	10
R middle frontal gyrus (R-MFG)	28.76	43	29	19	41	0.19	8
Relative Closeness × Emotion							
R TPJ (R-TPJ)	7.42	34	59	-36	26	0.13	40
Relative Closeness × Age							
Bilateral orbitofrontal cortex (B-OFC)	33.01	97	4	24	-11	0.14	11, 32
L inferior/middle temporal gyrus (L-ITG)	25.11	41	-54	-21	-16	0.19	21
R middle temporal gyrus (R-MTG)	24.63	29	67	-29	-11	0.12	21
Emotion							
Bilateral cerebellum and lingual gyrus	31.76	2670	-11	-49	-16	0.32	N/A, 18
L precentral/postcentral gyrus	30.38	1436	-31	-29	51	0.31	4
R precentral/postcentral gyrus	44.60	1402	41	-26	49	0.45	4
Bilateral medial frontal gyrus	18.32	639	-6	6	49	0.13	6
R inferior frontal gyrus	10.03	339	34	4	29	0.07	44
L insula	16.56	287	-29	24	6	0.17	13
R insula	14.00	212	34	21	4	0.13	13
R insula/postcentral gyrus	21.00	285	41	-21	19	0.27	1
L TPJ	9.03	167	-51	-51	31	0.12	39
R TPJ	10.53	136	49	-51	31	0.15	39
R superior temporal gyrus	8.67	85	49	-34	6	0.08	21
L superior parietal lobule	8.64	66	-29	-56	44	0.08	7
R thalamus	11.13	50	16	-19	4	0.16	N/A
L medial frontal gyrus	8.93	46	-6	-14	51	0.08	6
Sex × Emotion							
L lingual gyrus	7.73	69	-6	-96	-1	0.04	18


*Clusters listed here represent areas in which there were effects of Relative Closeness with peers, Age, Emotion, or their interactions on activation during stimulus presentation, controlling for Sex in the model. Clusters were formed using 3dclustsim at *p* < 0.001. Clusters of activation greater than the cluster size threshold of 27 voxels are presented here. There were no main effects of Age or Sex on activation. R, right; L, left; TPJ, temporal-parietal junction (e.g., supramarginal gyrus, angular gyrus, and inferior parietal lobule). *k*, cluster size in voxels. *xyz* coordinates represent the peak activation of the cluster, in Talairach-Tournoux space. η^*2*^, eta squared.*

**FIGURE 5:**
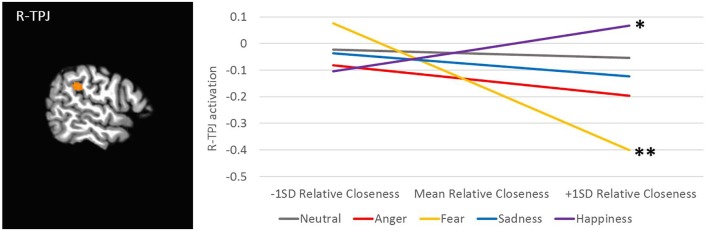
Interaction between participants’ relative closeness and facial emotion type on neural activation during stimulus presentation. R, right; TPJ, temporal-parietal junction. Cluster was formed using 3dclustsim at *p* < 0.001, with a cluster size threshold of 27 voxels. Brain image is rendered in the Talairach-Tournoux template space. Refer to Table 2 for description of region of activation. Plotted activation in the line graph is estimated from marginal means for activation by emotion type at low levels of Relative Closeness (–1 standard deviation), mean levels of Relative Closeness, and high levels of Relative Closeness (+1 standard deviation), with Sex and Age held constant at the mean. SD, standard deviation. The significance of the slope for different emotions (i.e., slope ≠ 0) is noted as ^∗^*p* < 0.05, ^∗∗^*p* < 0.01.

**FIGURE 6:**
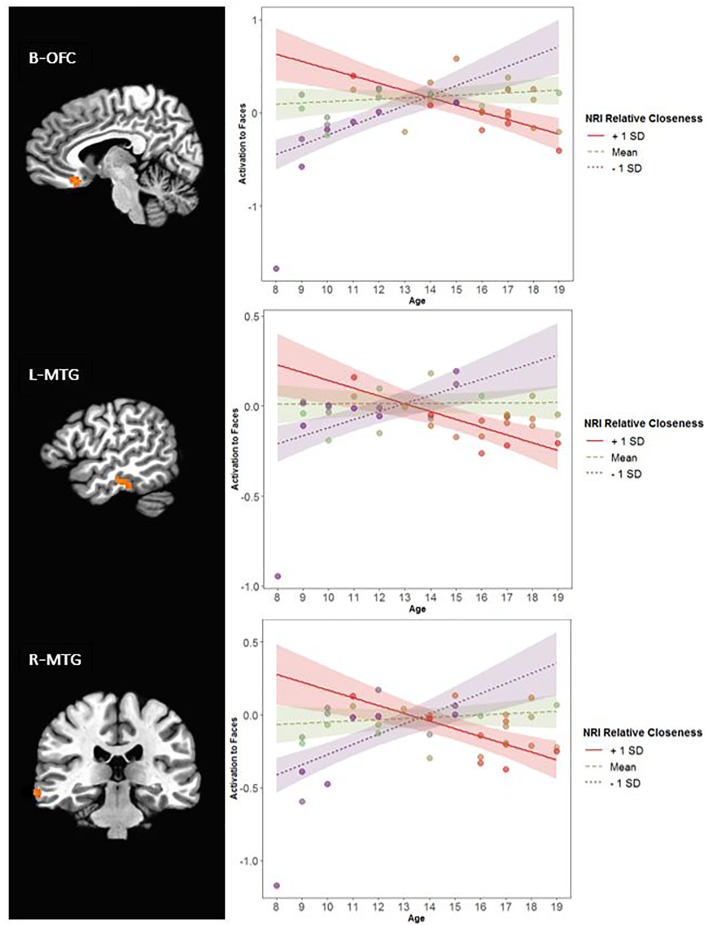
Interaction between participants’ relative closeness and age on neural activation during stimulus presentation. R, right; L, left; B, bilateral. OFC, orbitofrontal cortex; MTG, middle temporal gyrus. Clusters were formed using 3dclustsim at *p* < 0.001, with a cluster size threshold of 27 voxels. Brain images are rendered in the Talairach-Tournoux template space. Refer to Table 2 for description of regions of activation. Plotted activation in the line graphs represents estimated activation at low levels of Relative Closeness (–1 standard deviation), mean levels of Relative Closeness, and high levels of Relative Closeness (+1 standard deviation). SD, standard deviation. Colored bands surrounding the regression lines represent 95% confidence intervals. Of note, all interactions remained significant when the 8-year-old participant with low activation in these regions was removed.

Further, there was an interaction of Relative Closeness and Emotion in the right inferior parietal lobule and supramarginal gyrus (i.e., temporo-parietal junction, or R-TPJ; Table 2 and Figure 5). Parameter estimates for the effect of Relative Closeness on each emotion indicate that greater relative closeness with peers was associated with greater TPJ response to happy faces, *B* = 0.10, β = 0.41, *p* = 0.03, and lesser response to fearful faces, *B* = -0.28, β = -0.50, *p* < 0.01. Lastly, there was an interaction of Relative Closeness and Age in the bilateral orbitofrontal cortex at midline (B-OFC), the left inferior and middle temporal gyrus (L-MTG), and right middle temporal gyrus (R-MTG; Table 2 and Figure 6). In all these clusters, activation to peer-aged faces was greatest in younger participants who were relatively closer to peers than parents, and in older participants who were relatively closer to parents than peers. Activation to peer-aged faces was lowest in younger participants who were relatively closer to parents than peers and in older participants who were relatively closer to peers than parents.

### Functional Connectivity

To further understand their function in the context of the task, we conducted exploratory generalized psychophysiological interaction (gPPI) analyses (McLaren et al., 2012) to examine the functional connectivity of the two clusters in which a main effect of Relative Closeness with peers was noted (L-MFG and R-MFG). We first fit the same subject-level model to activation within those two regions of interest. We then performed a group-level model examining the effect of Age and Relative Closeness on functional connectivity with each of those seeds. Emotion and Sex were entered in the model as control variables. Identical cluster-size correction simulations were performed as above, with a resulting cluster threshold of 26 voxels.

**FIGURE 7:**
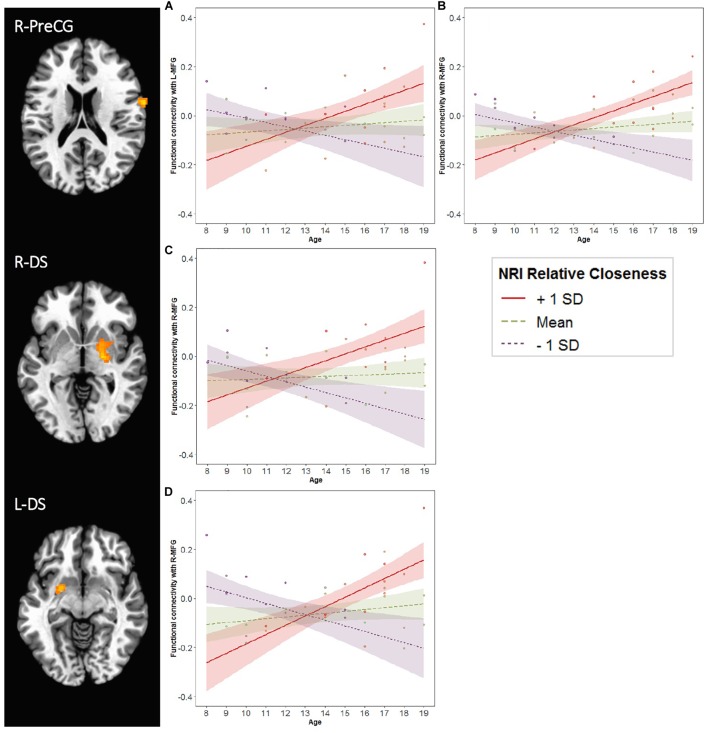
Age and Relative Closeness-related changes in functional connectivity with left and right middle frontal gyrus (MFG). Generalized psychophysiological interactions were computed by placing a seed in each of the two MFG clusters (L-MFG and R-MFG; see Table 2 and Figure 3). Brain regions above represent areas for which there was an interaction of Age × Relative Closeness on functional connectivity with the seeds. Clusters were formed using 3dclustsim at *p* < 0.001, with a cluster size threshold of 27 voxels. Refer to Table 3 for description of regions of activation. Brain images are rendered in the Talairach-Tournoux template space. L, left; R, right. PreCG, precentral gyrus; DS, dorsal striatum. The line graphs illustrate the Age × Relative Closeness on functional connectivity between the R-PreCG and L-MFG (graph **A**), the R-PreCG and R-MFG (graph **B**), the R-DS and R-MFG (graph **C**), and the L-DS and R-MFG (graph **D**). SD, standard deviation; NRI, Network of Relationships Inventory.

For both the L-MFG and R-MFG seeds, there was an Age × Relative Closeness interaction on functional connectivity with the right precentral gyrus (R-PreCG; Table 3 and Figure 7). In addition, there was also an Age × Relative Closeness interaction on functional connectivity between the R-MFG seed and both the right and left dorsal striatum (R-DS, L-DS; spanning the putamen and globus pallidus). Connectivity between seed regions and both the R-PreCG and dorsal striatum was strongest for older participants who were relatively closer to their peers than their parents. In contrast, a negative coupling between these regions was observed in younger participants who were closer to their peers, and in older participants who were closer to their parents.

**Table 3:** Generalized psychophysiological interaction analyses on functional connectivity with clusters of Relative Closeness-related activation.

						Generalized	Brodmann
Structure	*F*	*k*	*x*	*y*	*z*	η^2^	area
Seed in L-MFG							
R precentral gyrus	24.41	27	59	1	19	0.17	6
(R-PreCG)
Seed in R-MFG							
R precentral gyrus	37.87	35	64	-1	21	0.14	6
(R-PreCG)
R dorsal striatum (R-DS)	31.91	116	21	-11	-1	0.13	N/A
L dorsal striatum (L-DS)	26.42	28	-24	-1	-6	0.11	N/A


*Clusters listed here represent areas in which there was an interaction between Age and Relative Closeness on functional connectivity with the seed regions. Clusters were formed using 3dclustsim at *p* < 0.001. R, right; L, left. MFG, middle frontal gyrus. *k*, cluster size in voxels. *xyz* coordinates represent the peak activation of the cluster, in Talairach-Tournoux space. η^*2*^, eta squared. Some clusters were noted in the cerebellum (culmen) but are not noted here (available from first author). There were no main effects of Age, Relative Closeness, or Emotion on functional connectivity with either seed. A main effect of Sex on functional connectivity with the L-MFG seed was noted in the right insula, such that coupling between the two regions was greater for girls than boys (additional details available from first author).*

## Discussion

The current study examined age-related changes in 8- to 19-year-olds’ closeness with peers and parents, and investigated associations between relative closeness to peers and neural response to peer-aged facial expressions. Age was associated with increased relative closeness to peers over parents. Youth’s neural activation to teenage faces in frontal and temporal regions, as well as the functional connectivity between the dorsolateral prefrontal cortex (dlPFC) and the dorsal striatum (DS), depended on youth’s age and the extent of their orientation toward peers.

### Closeness With Peers and Parents

Though younger participants reported greater closeness with their parents than with their peers, older adolescents showed the opposite pattern. Age was associated with greater relative closeness with peers over parents; by mid-adolescence (approximately age 16), the majority of youth had arguably shifted toward reporting closer relationships with their friends than their caregivers. These results are consistent with an extensive body of work demonstrating changes in support, intimacy, interaction frequency, and complexity of parental and peer relationships during adolescence (Hunter and Youniss, 1982; Larson and Richards, 1991; Furman and Buhrmester, 1992; Rice and Mulkeen, 1995; Lieberman et al., 1999; De Goede et al., 2009). The enhanced salience of peers likely reflects evolutionarily conserved motivational mechanisms that guide attention and behavior toward greater social networks. Though positive family relationships in adolescence are important for social competence and other positive achievement outcomes (Bell et al., 1985; Field et al., 2002), close friendships take on a primordial role for teenagers (Steinberg and Morris, 2001; Foulkes and Blakemore, 2018). Teenagers spend most of their day in peer interactions (Crockett et al., 1984), and the importance of social bonds increases across adolescence: indeed, intimacy within friendships was more closely tied to adjustment and social competence in relationships in 13- to 16-year-olds than in 10- to 13-year-olds (Buhrmester, 1990). Establishing oneself within peer networks is a particularly important task for adolescents, and may buffer the negative impact of social stressors like rejection (Masten et al., 2010; Silk et al., 2011).

### Associations Between Relative Closeness With Peers and Neural Responses to Faces

As adolescents’ social networks broaden with age, neural networks underlying reward evaluation, response inhibition, and affective processing undergo continued development (Yurgelun-Todd, 2007). There is increasing recognition that variations in the peer environment can contribute to individual differences in neurocognitive processing of social and emotional stimuli (Foulkes and Blakemore, 2018). In the framework of the SIPN model of adolescents’ social and neural development, our hypothesis was that greater orientation toward peers (i.e., greater relative closeness with peers than with parents) would be associated with increased response in reward- or affect-related nodes of the brain, but reduced activation in cognitive-regulatory regions. Results suggest that neural activation in, and functional connectivity between, these nodes varies with both relative closeness with peers and its interaction with age.

Contrary to our hypothesis, we did not find evidence that greater closeness with peers was associated with differential response in traditional affect-related regions of the brain, such as the amygdala or ventral striatum. However, individuals who reported greater relative closeness with peers over parents (collapsed across age) showed less activation in the dlPFC (i.e., R-MFG and L-MFG) than those who reported greater closeness with parents. Regions of the dlPFC have been implicated in many higher-order functions, such as working memory (e.g., Nelson et al., 2000; Cole and Schneider, 2007), decision-making (including risk-taking; e.g., Krain et al., 2006; Rao et al., 2008), emotion regulation (Golkar et al., 2012), and attentional or cognitive control (MacDonald et al., 2000; Cole and Schneider, 2007; Kompus et al., 2009; Kohn et al., 2014). The experimental paradigm we employed does not enable us to determine the precise function of the dlPFC in this task. However, the R-MFG and L-MFG clusters that varied by relative closeness with peers (located approximately in Brodmann areas 8 and 10) have been involved in the up- and down-regulation of emotional response (Li et al., 2018), impulse control in delay discounting tasks (Weygandt et al., 2015), the selection of “safe” choices in risk-taking paradigms (Chein et al., 2011; Crowley et al., 2015; Van Leijenhorst et al., 2010), and response inhibition in go-no-go (Li et al., 2006; Chikazoe et al., 2009) or Stroop tasks (Aarts et al., 2009). In the current study, it is possible that reduced activation in these regions is reflective of lessened inhibitory control responses to novel teenage faces – a pattern that would be expected in youth who were relatively closer to their peers than their parents. Alternatively, youth who are closer with peers may not need to engage as many emotion regulation or effortful control resources when responding to the facial expressions of peer-aged teenagers.

Though these interpretations are speculative and cannot be formally tested in the current study, functional connectivity analyses support the hypothesized inhibitory or regulatory function of the dlPFC. The coupling between both dlPFC seed regions and either the right precentral gyrus or the DS varied by participant age and their relative closeness with peers. Inhibitory control processes are thought to be mediated by a fronto-basal ganglia circuit (for reviews, see Verbruggen and Logan, 2008; Chikazoe, 2010) encompassing ventral and dorsal prefrontal regions and the globus pallidus in the DS (Aron and Poldrack, 2006; Dillon and Pizzagalli, 2007). Moreover, the DS itself has been found to contribute to aspects of reward processing and goal-directed action. Activation in the DS has been elicited by both reward and punishment (e.g., Bjork et al., 2004; Delgado, 2007; Münte et al., 2017), as well as the anticipation of rewards (e.g., Knutson et al., 2001; Spreckelmeyer et al., 2009). Further, the DS is thought to be implicated in the association between stimuli, actions, and rewards (O’Doherty, 2004; Haruno and Kawato, 2006; Balleine et al., 2007) and the encoding of the value of different outcomes (Delgado et al., 2003) in the context of reward-based learning.

Developmental neuroscience theories of adolescence have highlighted the “mismatch” in the timing of maturation between early-developing subcortical structures (including the striatum) and later-developing neocortical structures during the teenage years (Steinberg, 2005; Casey et al., 2011). Poor prefrontal regulatory influence on affect- or reward-related subcortical areas has been proposed to contribute to many phenotypic aspects of adolescence (e.g., Nelson et al., 2016; Shulman et al., 2016), including the heightened motivational salience of peers (Nelson and Guyer, 2011; Schriber and Guyer, 2016). In our sample, functional connectivity between the dlPFC and DS regions was strongest for older youth who were closer to their peers – those who, it may be argued, reported the developmentally expected patterns of orientation toward friends. In contrast, for youth who did not follow this pattern (and who were either closer to peers at a young age, or closer to parents in their late adolescence), there was a negative coupling between the dlPFC and the DS. Thus, younger youth who were closer to their peers showed lower dlPFC and greater DS activation in response to peer-aged faces, whereas older youth who were closer to their parents showed greater dlPFC and lower DS activation. It is possible that differences in connectivity for younger participants may be driven by immature structural connections between frontal and striatal regions; however, the presence of a similar pattern for older adolescents suggests that variations in brain structure are not sufficient to explain these findings. Alternatively, these respective neural patterns may be associated with the facilitation of orientation toward peers (low inhibitory control paired with high response in valuation-related regions) or the hindrance of this behavioral tendency (high inhibitory control and low valuation response). This interpretation is strictly hypothetical, though it is in line with theoretical predictions about the interplay of changes in social behavior, the salience of peers, and the interaction of affective and cognitive-regulatory nodes of the brain (Nelson et al., 2016). To test this hypothesis, future studies should explore how social re-orientation is associated with dlPFC and DS activation in tasks that explicitly assess reward processing and inhibitory control in response to peer-aged social cues.

Further, the association between relative closeness with peers and neural activation to faces in several regions of the social brain was found to vary depending on either (a) stimulus emotion, or (b) participant age. Emotion-specific differences in closeness-related activation were found in the right TPJ, an area heavily involved in social cognitive functions like the perception and interpretation of others’ affect and beliefs (Saxe and Wexler, 2005; Van Overwalle, 2009). Specifically, youth who were closer to their peers than their parents (regardless of their age) showed greater activation to happy faces, and less activation to fearful faces, in the TPJ. This finding is consistent with past work indicating that 14- to 18-year-olds who reported greater emotional closeness with their peers showed heightened TPJ response to social reward (Flores et al., 2018). Happy faces are generally considered to be rewarding social cues, whereas fearful faces may be aversive or socially threatening. Elevated TPJ response to positive social cues and reduced response to negative cues may underlie a tendency to recruit mentalizing networks more in the context of social approach signals, which may facilitate positive mutual engagement with peers.

Age-related variations in the association between relative closeness and brain activation were also noted in the orbitofrontal cortex (OFC) and the temporal lobes. Greater activation in these brain regions was noted for younger participants who were closer to their peers than parents, and older youth who were closer to their parents. The temporal lobes are extensively involved in multimodal and affective integration of social stimuli (Zilbovicius et al., 2006; Morin et al., 2014; Pitcher et al., 2017), while the medial portions of the OFC are generally implicated in valuation and reward (O’Doherty et al., 2001; Roelofs et al., 2008; Leppanen and Nelson, 2009; Murray and Wise, 2010). In the present context, this pattern of activation may indicate enhanced value and integrative processing of peer stimuli in young adolescents who are particularly drawn to their peers, but also in older adolescents and young adults who have not developed close bonds with their friends. Though speculative, it is possible that the increased activation in the above social brain regions reflects increased valuation of peer-aged cues for these two groups of teenagers who must either continue to orient toward peers or begin to do so.

### Strengths and Limitations

To our knowledge, this is the first study that examines associations between youth’s social orientation toward peers (i.e., emotional closeness with friends compared to parents) and their neural response to peer-aged facial expressions of emotion. Though the current study did not assess social behaviors with peers, results highlight potential neural markers of social re-orientation that may either accompany or facilitate behavioral approach toward peers during the teenage years (Nelson et al., 2016). However, limitations must be noted. First, we used youth’s relative closeness to peers compared to their closeness with their parents as a proxy for social orientation tendencies; future studies will need to supplement this estimate of social development with objective measures of social experiences and behaviors, such as those obtained with ecological momentary assessment paradigms. Second, the current study only evaluated youth’s neural response to peer-aged faces. A more stringent test of our hypothesis that individual variations in social orientation are associated with differential neural response to peer-aged cues requires the inclusion of adult faces as a comparison condition. The use of individualized stimuli obtained from participants’ own friends and parents would have also provided more specific information about the neural representation of social experiences in close relationships. Though adolescents’ processing of unfamiliar peers’ faces is relevant to the process of integrating with novel social groups during the teenage years, future work would benefit from the use of personally relevant stimuli in experimental paradigms assessing social cognition.

Third, the current study cannot pinpoint the extent to which changes in emotional closeness with others and neural responses to emotional faces are due to variations in adrenarcheal or gonadal hormones (e.g., Whittle et al., 2015). Pubertal status, as well as the timing and tempo of pubertal development, are thought to play a large role in psychological and neural functioning (Angold et al., 1998; Lenroot and Giedd, 2010; Byrne et al., 2017). Though age and pubertal status are highly correlated, the assessment of pubertal maturation would add to our understanding of developmental changes in both social behavior and the neural processing of facial stimuli. Replication in a larger sample size would also strengthen our conclusions about age-related changes in brain activation patterns across late childhood and adolescence. Lastly, the present design does not enable tests of directionality. As individual differences in peer environments may influence neural response to social stimuli, so may individual differences in neurobiology affect adolescents’ social behaviors and sensitivity to socio-emotional cues (Foulkes and Blakemore, 2018). Additional work in longitudinal frameworks would help clarify the association between neural response and social experiences in adolescence.

## Conclusion

Adolescence is characterized by a myriad of changes in body, brain, and behavior. Among these transitions, the teenage years are marked by a social re-orientation toward peers – a process that is likely bolstered and accompanied by changes in how social stimuli from other adolescents are valuated and processed neurally. The results of the current study suggest that individual differences in teenagers’ peer experiences (denoting social re-orientation toward friends, or a lack thereof) are associated with differential brain responses to peer-aged faces. Age was associated with greater relative closeness to peers than to parents, which can be conceptualized as a marker of having achieved the transition toward a broader peer network. Across all ages, greater relative closeness to peers itself was related to (a) lessened activation in frontal regions associated with inhibitory or regulatory functions, (b) reduced response to fearful social cues in the TPJ, and (c) greater response to positive social cues in the TPJ. In addition, both activation within regions of the social brain (orbitofrontal cortex, temporal lobes), and functional connectivity between dorsolateral prefrontal cortex and the dorsal striatum, varied as a function of youth’s age and closeness to peers. Specifically, both increased activation in frontal and temporal regions involved in the evaluation of socio-emotional stimuli, and negative coupling between the dlPFC and DS, were noted in early adolescents who had transitioned toward peers, and late adolescents who had failed to do so. Though replication with extended study designs will be necessary, such neural response to peer-aged cues may support the positive valuation of peers that may be necessary to encourage motivational tendencies toward peer interactions.

In conclusion, engaging with peers and forming close social bonds is a crucial developmental task, which may be accompanied by changing neural response to peers’ social signals in social cognitive, inhibitory control, and reward-related networks. Understanding the normative interrelated changes to neural systems and social behavior in adolescence is necessary for the characterization of typical developmental trajectories and deviations from those norms in teenagers who struggle to form meaningful peer relationships.

## Data Availability

The datasets generated for this study are available on request to the corresponding author.

## Ethics Statement

This study was carried out in accordance with the recommendations of the Institutional Review Board of the Research Institute at Nationwide Children’s Hospital with written informed consent from all subjects. All subjects gave written informed consent in accordance with the Declaration of Helsinki. The protocol was approved by the Institutional Review Board.

## Author Contributions

All authors contributed to the study design, data collection, statistical analysis, and manuscript preparation.

## Conflict of Interest Statement

The authors declare that the research was conducted in the absence of any commercial or financial relationships that could be construed as a potential conflict of interest. The handling Editor declared a past co-authorship with one of the authors EN.

## Footnotes

1 Due to scanner updates during data collection, five participants were tested on a different scanner than the other 35 participants. Results were highly similar to those presented in the manuscript when these participants were excluded from analyses (see Supplementary Table 1 in Supplementary Materials).

2 There were age effects on the amount of motion during the scan, such that age was negatively related to the fraction of censored volumes per participant (β = -0.43, *p* = 0.006). Including participants’ fraction of total volumes censored as a covariate did not alter the results presented here (with the exception of the Age × Relative Closeness effect on the R-MTG cluster, which was just below cluster correction thresholds for size at 26 voxels).
