# Supplementary material for: Associations Between Adolescents’ Social Re-orientation Toward Peers Over Caregivers and Neural Response to Teenage Faces
Source: Front Behav Neurosci. 2019 May 24;13:108. doi: 10.3389/fnbeh.2019.00108 (PMC6544008; doi:10.3389/fnbeh.2019.00108)
Supplement: Supplementary file 1 [file Table_1.DOCX]

**Supplemental Materials**

The following tables represent results for analyses relating to neural activation, when removing the 5 participants who were scanned on a different scanner/head-coil. Table 1 lists results for the multivariate linear model (MVM) analysis. Table 2 lists results for the generalized psychophysiological interaction (gPPI) analyses exploring functional connectivity with the R-MFG and L-MFG clusters in Table 1. Results are generally consistent with results from the original full sample, with a few exceptions. First, new clusters of significance were found for the main effect of Age, the main effect of Relative Closeness, and the interaction of Age and Relative Closeness. Second, a few of the original clusters reported are now below cluster correction thresholds (*k* = 27 for multivariate model; *k* = 26 for gPPI models) in size. Specifically, the right middle temporal gyrus (R-MTG) cluster of significance for Age x Relative Closeness in the MVM is now 18 voxels. For gPPI analyses, the right precentral gyrus (R-PreCG) cluster that was functionally connected to the L-MFG seed, and the left dorsal striatum (L-DS) cluster that was functionally connected to the R-MFG seed, are now 14 and 25 voxels, respectively.

Table 1

Effects of Relative Closeness, Age, and Emotion on neural activation to faces, without participants scanned with different head-coil

| Effect  Structure | *F* | *k* | *x* | *y* | *z* | Brodmann area |
| --- | --- | --- | --- | --- | --- | --- |
| Age |  |  |  |  |  |  |
| L precentral gyrus | 26.74 | 93 | -29 | -16 | 59 | 6 |
| R middle frontal gyrus | 24.57 | 40 | 34 | 19 | 34 | 9 |
| L inferior parietal lobule | 20.28 | 33 | -31 | -44 | 54 | 40 |
| Relative Closeness |  |  |  |  |  |  |
| Bilateral anterior cingulate | 34.10 | 250 | 6 | 29 | 16 | 32 |
| Bilateral medial frontal gyrus | 21.05 | 128 | 1 | 39 | 34 | 9 |
| R middle frontal gyrus (R-MFG) | 35.33 | 93 | 29 | 19 | 41 | 8 |
| L middle frontal gyrus (L-MFG) | 32.38 | 49 | -39 | 49 | 11 | 10 |
| Relative Closeness x Emotion |  |  |  |  |  |  |
| R TPJ (R-TPJ) | 8.7 | 52 | 59 | -36 | 26 | 40 |
| Relative Closeness x Age |  |  |  |  |  |  |
| Bilateral orbitofrontal cortex (B-OFC)† | 39.18 | 113 | 2 | 21 | -20 | 11 |
| L inferior/middle temporal gyrus (L-ITG) | 21.90 | 32 | -54 | -21 | -16 | 20 |
| L temporal pole | 25.29 | 39 | -39 | -11 | -31 | 20 |
| L postcentral gyrus | 25.29 | 75 | -36 | -36 | 54 | 1 |
| L superior occipital gyrus | 37.18 | 31 | -41 | -79 | 31 | 19 |
| Emotion |  |  |  |  |  |  |
| R cerebellum & lingual gyrus | 11.92 | 655 | 21 | -71 | -6 | N/A, 18 |
| L cerebellum & lingual gyrus | 13.77 | 574 | -16 | -79 | -9 | N/A, 18 |
| L precentral/postcentral gyrus | 26.34 | 609 | -31 | -29 | 54 | 4 |
| R precentral/postcentral gyrus | 40.39 | 992 | 36 | -31 | 51 | 4 |
| L inferior frontal gyrus/precentral gyrus | 14.57 | 288 | -39 | 4 | 31 | 6, 9 |
| R inferior frontal gyrus/precentral gyrus | 7.68 | 50 | 34 | 4 | 29 | 6, 9 |
| R insula | 14.79 | 151 | 44 | -19 | 19 | 13 |
| L insula | 10.5 | 101 | -29 | 24 | 6 | 13 |
| L culmen | 22.33 | 129 | -11 | -49 | -16 | N/A |
| Bilateral medial frontal gyrus | 11.38 | 121 | -6 | 6 | 49 | 6 |
| R TPJ | 7.61 | 49 | 49 | -51 | 31 | 39 |
| L precentral gyrus | 8.43 | 40 | -26 | -9 | 49 | 6 |
| R superior temporal gyrus | 7.76 | 35 | 46 | -39 | 9 | 22 |
| R medial frontal gyrus | 9.17 | 34 | 9 | -16 | 49 | 6 |
| L middle frontal gyrus | 7.49 | 30 | -44 | 49 | 16 | 10 |

*Note.* Clusters listed here represent areas in which there were effects of Relative Closeness with peers, Age, Emotion, or their interactions on activation during stimulus presentation, controlling for Sex in the model. Clusters were formed using 3dclustsim at *p* < .001. Clusters of activation greater than the cluster size threshold of 27 voxels are presented here. There was no main effect of Sex on activation. R = right, L = left. TPJ = temporal-parietal junction (e.g., supramarginal gyrus, angular gyrus, and inferior parietal lobule). *k* = cluster size in voxels. *xyz* coordinates represent the peak activation of the cluster, in Talairach-Tournoux space. † = coordinates represent the cluster’s center of mass, as the peak was on the edge of the brain.

Table 2

Generalized psychophysiological interaction analyses on functional connectivity with clusters of Relative Closeness-related activation, without participants scanned with different head-coil

| Structure | *F* | *k* | *x* | *y* | *z* | Brodmann area |
| --- | --- | --- | --- | --- | --- | --- |
| Seed in L-MFG |  |  |  |  |  |  |
| - |  |  |  |  |  |  |
| Seed in R-MFG |  |  |  |  |  |  |
| R precentral gyrus (R-PreCG) | 35.73 | 27 | 64 | -1 | 21 | 6 |
| R dorsal striatum (R-DS) | 31.97 | 141 | 26 | -11 | -4 | N/A |

*Note.* Clusters listed here represent areas in which there was an interaction between Age and Relative Closeness on functional connectivity with the seed regions. Clusters were formed using 3dclustsim at *p* < .001. R = right, L = left. MFG = middle frontal gyrus. *k* = cluster size in voxels. *xyz* coordinates represent the peak activation of the cluster, in Talairach-Tournoux space. There were no main effects of Age, Relative Closeness, or Emotion on cortical functional connectivity with either seed. Some clusters were noted in the cerebellum but are not noted here (available from first author). A main effect of Sex on functional connectivity with the L-MFG seed was noted in the right insula, such that coupling between the two regions was greater for girls than boys (additional details available from first author).
