# Supplementary material for: Associations Between Adolescents’ Social Re-orientation Toward Peers Over Caregivers and Neural Response to Teenage Faces
Source: Front Behav Neurosci. 2019 May 24;13:108. doi: 10.3389/fnbeh.2019.00108 (PMC6544008; doi:10.3389/fnbeh.2019.00108)
Supplement: Supplementary file 2 [file Table_2.docx]

Appendix

List of NIMH-ChEFS images used in current study

F10HA_4379.jpg

F10HS_4372.jpg

F16HS_5924.jpg

F3HA_3545.jpg

F3HS_3540.jpg

F4HA_3668.jpg

F4HS_3664.jpg

F8HA_4091.jpg

F8HS_4086.jpg

M11HA_6890.jpg

M14HA_7913.jpg

M14HS_7912.jpg

M19HA_9066.jpg

M2HS_4493.jpg

M4HA_4877.jpg

M4HS_4862.jpg

M9HA_6057.jpg

M9HS_6054.jpg

F14FA_5638.jpg

F14FS_5632.jpg

F19FS_6344.jpg

F1FS_3388.jpg

F2FA_3491.jpg

F2FS_3470.jpg

F37FA_9020.jpg

F4FA_3712.jpg

F4FS_3704.jpg

M10_4FS_6817.jpg

M11FA_6969.jpg

M15FA_8374.jpg

M15FS_8364.jpg

M19FA_9129.jpg

M19FS_9117.jpg

M3FA_4789.jpg

M3FS_4775.jpg

M4FA_4941.jpg

F1AS_3411.jpg

F31AA_7892.jpg

F31AS_7888.jpg

F38AA_9403.jpg

F38AS_9388.jpg

F7AA_4061.jpg

F7AS_4050.jpg

F9AA_4268.jpg

F9AS_4247.jpg

M14AA_7977.jpg

M14AS_7973.jpg

M15AS_8385.jpg

M16AA_8705.jpg

M18AA_8933.jpg

M18AS_8919.jpg

M19AA_9164.jpg

M7AA_5567.jpg

M7AS_5550.jpg

F13SA_5284.jpg

F13SS_5276.jpg

F19SS_6316.jpg

F36SA_8543.jpg

F36SS_8532.jpg

F6SA_3901.jpg

F6SS_3889.jpg

F7SA_4013.jpg

F7SS_4001.jpg

M10SA_6795.jpg

M10SS_6781.jpg

M14SA_7945.jpg

M14SS_7930.jpg

M16SA_8665.jpg

M16SS_8654.jpg

M3SA_4746.jpg

M3SS_4742.jpg

M9SA_6092.jpg

F18NS_6177.jpg

F19NA_6296.jpg

F25NS_5531.jpg

F30NA_8493.jpg

F30NS_8489.jpg

F5NA_3751.jpg

F5NS_3749.jpg

F7NA_3982.jpg

F9NA_4182.jpg

F9NS_4175.jpg

M11NA_6880.jpg

M16NA_8630.jpg

M16NS_8625.jpg

M18NA_8847.jpg

M20NA_9185.jpg

M20NS_9182.jpg

M4NS_4853.jpg

M7NS_5485.jpg
